# A novel *UBA1* gene mutation in a patient with infantile respiratory distress syndrome

**DOI:** 10.1038/s41439-024-00307-7

**Published:** 2025-01-06

**Authors:** Masafumi Miyata, Arisa Kojima, Yuri Kawai, Hidetoshi Uchida, Hiroko Boda, Naoko Ishihara, Hidehito Inagaki, Tetsushi Yoshikawa, Hiroki Kurahashi

**Affiliations:** 1 Department of Pediatrics, Fujita Health University School of Medicine, Toyoake, Aichi Japan; 2 Division of Molecular Genetics, Center for Medical Science, Fujita Health University Hospital, Toyoake, Aichi Japan; 3 Department of Clinical Genetics, Fujita Health University Hospital, Toyoake, Aichi Japan

**Keywords:** Disease genetics, Mutation

## Abstract

UBA1 is an E1 ubiquitin-activating enzyme that initiates the ubiquitylation of target proteins and is thus a key component of the ubiquitin signaling pathway. Three disorders are associated with pathogenic variants of the *UBA1* gene: vacuoles, E1 enzyme, X-linked, autoinflammatory, somatic (VEXAS) syndrome, lung cancer in never smokers (LCINS), and X-linked spinal muscular atrophy (XL-SMA, SMAX2). We here report a case of infantile respiratory distress syndrome followed by continuing neuromuscular symptoms. We identified a de novo hemizygous mutation, c.1660 C > T (p.Pro554Ser), in exon 15 of the *UBA1* gene in this baby. This missense mutation was located with the AAD (active adenylation domain) of the protein, a known hotspot of SMAX2 mutations. This case lends support to the genotype-phenotype correlation regarding the *UBA1* mutation and its related diseases.

Ubiquitylation is a three-step process involving the concerted actions of the E1 ubiquitin-activating enzyme, E2 ubiquitin-conjugating enzyme, and E3 substrate-specific ligase. The *UBA1* gene encodes the enzyme initiating the first step of protein ubiquitylation that leads to proteolysis via the ubiquitin-proteasome pathway. A conventional positional cloning strategy was used previously to map the responsible locus for X-linked spinal muscular atrophy (XL-SMA, SMAX2) to Xp11.3-Xq11.2, a region that includes the *UBA1* gene in which germline mutations were identified in SMAX2 patients^1^. Recently, somatic mutations of the *UBA1* gene were identified in patients with vacuoles, E1 enzyme, X-linked, autoinflammatory, somatic (VEXAS) syndrome in males and lung cancer in never-smokers (LCINS) in females^2,3^. Although the mutations identified in SMAX2 patients are located exclusively in exon 15, those identified in VEXAS or LCINS are outside of the SMAX2 mutation hotspot, and the principle of this genotype-phenotype correlation thus remains in question^4^. In this present case report, we describe an SMAX2 patient harboring a *UBA1* mutation at exon 15, thereby providing supportive evidence for the genotype-phenotype correlation for this mutation and its related diseases

The study patient was a male born by cesarean section at an obstetrics clinic at 37 weeks and 1 day of gestation due to a breech presentation. His mother and father were 30 and 26 years of age, respectively. His mother’s reproductive history included a gravidity of 2 and parity of 1 and no assisted reproductive technology. His 3-year-old sister had B-cell precursor lymphoblastic leukemia. The patient’s birth weight, height, and head circumference were 2890 g, 47 cm, and 34 cm, respectively.

The Apgar score for this case was 5 points at 1 min after birth and 1 point at 5 min after birth. The patient was resuscitated by intubation and admitted to our neonatal intensive care unit. A subsequent chest X-ray revealed a reticulo-granular pattern, and a stable microbubble test was weak. The patient was subsequently diagnosed with respiratory distress syndrome (RDS) and administered surfactant. Upon respiratory improvement, he was extubated at one day of age. His respiration had worsened at day 2 days of age, however, and he was re-intubated and again treated with surfactant. In addition to this, nitric oxide inhalation was performed at 6 days of age due to persistent pulmonary hypertension. The patient’s respiration then gradually improved and he was re-extubated and received continuous nasal positive airway pressure at 8 days of age. His respiratory support was changed to a high-flow nasal cannula at 12 days of age and was ceased at 14 days of age. Enteral nutrition had commenced by tube feeding at 5 days of age, and oral feeding had started at 13 days of age. The respiratory status eventually reached stability and oral feeding was good, and the patient was discharged at 37 days of age. To test for possible congenital pulmonary alveolar proteinosis, a blood sample taken at 11 days of age was submitted for targeted exome analysis of the *SFTPB*, *SFTPC* and *ABCA3* genes, but no significant mutations were detected.

Outpatient follow-up of the patient’s growth and development reported a social smile at 2 months of age. However, a motor developmental delay was later observed. The patient could hold his head up at 5 months and roll over at 7 months. At 1 year and 1 month, he could sit with assistance in adjusting his posture and raise his chest with both hands in the supine position. At 1 year and 6 months, he could speak a few words but was unable to crawl. At 2 years of age, his speech was limited to connecting two words only, and he could crawl a little and stand on his knees. There were no problems with the movement of his fingers, and he was able to hold the food in his own hands. No elevations in muscle deviation enzymes (AST, LDH, aldolase) were observed during the clinical course. When we looked back and checked the medical records, the infant was receiving respiratory rehabilitation when he was ventilated, and the doctor of rehabilitation medicine had recorded the presence of a grasping reflex and the stiffness of the knee joint without an increased muscle tone in the medical record at 7 days of age. At 2 years old, the deep tendon reflexes of the upper limbs were present, but the deep tendon reflexes of the lower limbs could not be observed. Central nervous systems image screening tests included a brain MRI at 34 days of age and at 1 year and 5 months, and a spine MRI (Fig. 1) at 1 year and 11 months, but showed no abnormal findings. The exome data were reanalyzed at 2 years and 2 months of age to elucidate the pathogenesis of his floppy infant symptoms after recovery from respiratory distress syndrome in the neonatal period.

**Fig. 1:**
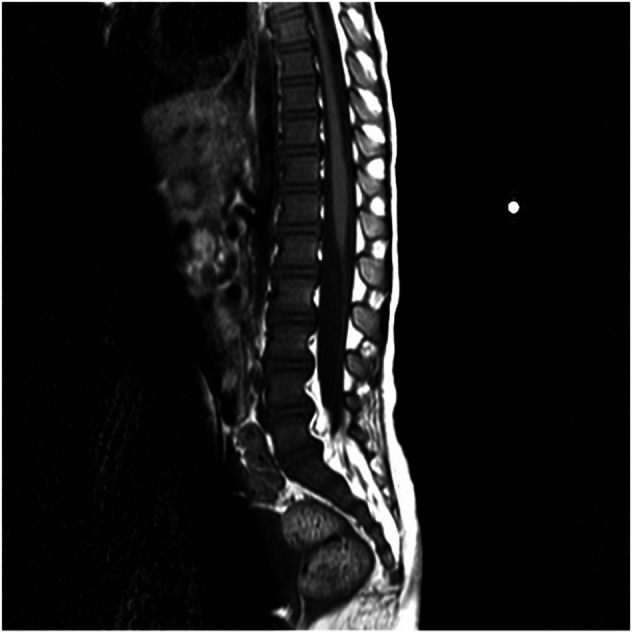
T1-weighted sagittal image of the spinal cord MRI. No abnormal findings were observed.

We performed targeted exome sequencing for the capture of 4813 disease-associated genes (Illumina, San Diego, CA). We then screened these data using RDS as a keyword, but no mutation was identified among the candidate genes. We reanalyzed the exome data for our current case and identified a missense variant, NM_003334: c.1660 C > T (p.Pro554Ser), in exon 15 of the *UBA1* gene. This was confirmed by Sanger sequencing (Fig. 2A). We then analyzed parental samples and found that this was a de novo variant that was not present in any databases of normal healthy populations such as ExAC. This variant is located at exon 15 of the *UBA1* gene, a known hotspot of SMAX2 mutations^1,5–9^. The Pro554 residue is a highly conserved amino acid among various species (human, rhesus monkey, mouse, dog, elephant, chicken and zebrafish) within the conserved AAD (active adenylation domain) that constitutes a putative hotspot of SMAX2 missense mutations in the UBA1 protein (Fig. 2B, C). In silico functional analysis predicted, however, that the p.Pro554Ser variant was benign when using BeyesDel (-0.2915 addAF score, -0.6564 noAFscore) and also with MetaRNN (0.2221). Taken together, these data indicated that the variant was likely pathogenic in accordance with the ACMG-AMP criteria (PM1, PM2, PM6, PP4).

**Fig. 2:**
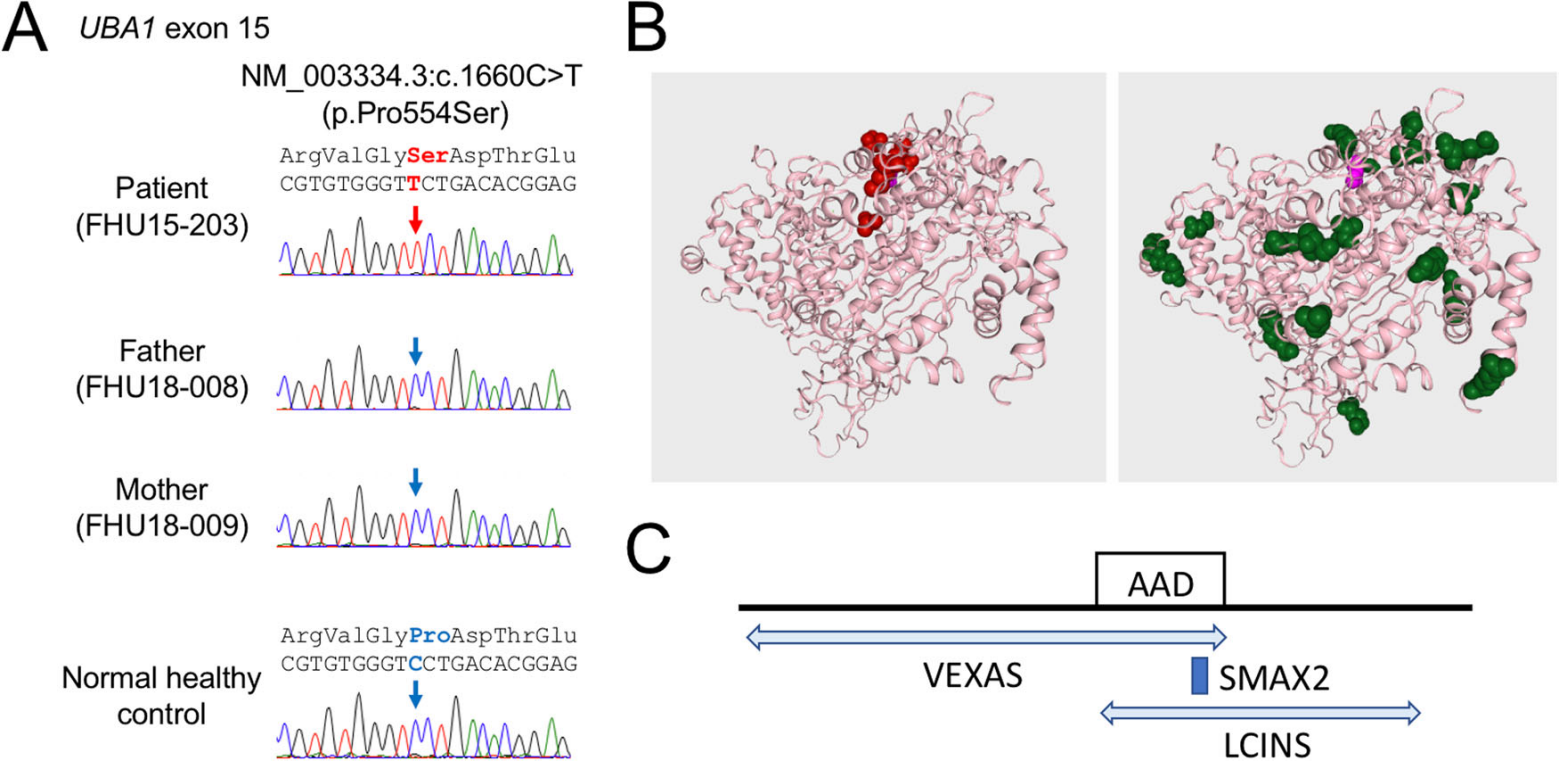
Mutational analysis of the current study patient presenting with the XL-SMA, SMAX2 disorder. **A** Sanger sequencing analysis of the patient, patient’s father and mother, and healthy control. A de novo hemizygous NM_003334: c.1660 C > T (p.Pro554Ser) mutation was identified in the *UBA1* gene. **B** Prediction of UBA1 protein structures generated by 3D Protein Viewer in VarSome using SwissModel:6dc6 49-1057. The reported SMAX2 mutations (red) are clustered in a small region of the 3D structure (left). The p.Pro554Ser mutation (pink) located at the surface of the protein is unlikely to affect the protein structure significantly, but could potentially affect the binding to its partner protein (right). The green colour denotes previously reported benign variants. **C** The *UBA1* gene mutation identified in the study patient was located in exon 15 and affected the conserved AAD domain in the UBA1 protein. This region is a known hotspot of SMAX2 missense mutations (dark blue box). Somatic VEXAS mutations and LCINS mutations are more widespread in the *UBA1* gene (light blue arrows).

The AAD domain interacts with gigaxonin, a member of the BTB/kelch superfamily, which forms complexes with UBA1 to regulate MAPB1, a ubiquitin-related microtubule-associated protein that functions in neurodevelopment and neurodegeneration^10^. The UBA1 mutant protein has previously been reported to have reduced ubiquitin-adenylating activity and defects in the transfer of ubiquitin to the E2 enzyme, possibly leading to adverse neurodevelopmental effects^4,11^. On the other hand, the synonymous variant c.1731 C > T (p.Asn577Asn) located in exon 15 of the *UBA1* gene has been reported in both familial or de novo SMAX2 cases, suggesting that this region plays a pivotal role at the nucleotide level^1,6,9^. Notably, the use of Splice AI does not predict a splicing enhancer or suppressor within this region. Since *UBA1* exon 15 harbors CpG islands (13 CpGs) and C > T synonymous variants correspond with the CpG nucleotides that are methylated, it has been proposed that these C > T mutations may inhibit protein binding and thus lead to modification of *UBA1* gene expression^1^. Of note in this regard, the c.1660 C > T substitution does not affect these CpGs, but c.1660 C is highly conserved among species even at the nucleotide level (Fig. 2C), leaving the possibility that it is also located within the potential protein binding site and thus may disrupt UBA1 protein function at the nucleotide level.

Since SMAX2 is a disorder that only affects males, it is likely that the SMAX2 variant is a loss-of-function (LOF) type. No truncation mutation or whole gene deletion has been identified, suggesting that a complete loss of UBA1 activity may be embryonically lethal^12^. Similarly, VEXAS syndrome only arises in males who carry a somatic LOF variant in the *UBA1* gene. LOF variants in VEXAS appear within a larger region in the gene than SMAX2 mutations (Fig. 2C), possibly because a LOF mutation can be tolerated at the cellular level^4^. In contrast to this, LCINS mutations may behave as driver mutations in a similar manner to autosomal tumor suppressor genes since the *UBA1* gene escapes X chromosome inactivation^3^. LOF variants in LCINS also appear within a larger region in the *UBA1* gene than SMAX2 mutations for similar reasons^4^.

Finally, to confirm the diagnosis of SMAX2, an electromyogram was performed at 6 years of age. The amplitude of the compound motor action potential all decreased; peak-to-peak amplitudes were 1.55 mV and 1.90 mV at the wrist and elbow in the median nerve, 0.44 mV and 0.59 mV at the wrist and elbow in the ulnar nerve, and 0.39 mV and 0.26 mV at the ankle and popliteal fossa in tibial nerve. These findings are consistent with the diagnosis of SMAX2.

In summary, SMAX mutations are located within the small exon 15 region in the *UBA1* gene, and the c.1660 C > T (p.Pro554Ser) germline mutation identified in our present case study is a strong candidate as the cause of this patient’s phenotype.

## HGV database

The relevant data from this Data Report are hosted at the Human Genome Variation Database at 10.6084/m9.figshare.hgv.3479.
